# Conceptualizing a Sustainable Food System in an Automated World: Toward a “Eudaimonian” Future

**DOI:** 10.3389/fnut.2018.00104

**Published:** 2018-11-05

**Authors:** Alon Shepon, Patrik John Gustav Henriksson, Tong Wu

**Affiliations:** ^1^Department of Plant and Environmental Sciences, Weizmann Institute of Science, Rehovot, Israel; ^2^Stockholm Resilience Centre, Stockholm University, Stockholm, Sweden; ^3^WorldFish, Jalan Batu Maung, Malaysia; ^4^The Beijer Institute of Ecological Economics, The Royal Swedish Academy of Sciences, Stockholm, Sweden; ^5^School of Life Sciences, Arizona State University, Tempe, AZ, United States

**Keywords:** automation, food system, food security, agroecology, social-ecological system, Eudaimonian food system

## Abstract

The industrialized world has entered a new era of widespread automation, and although this may create long-term gains in economic productivity and wealth accumulation, many professions are expected to disappear during the ensuing shift, leading to potentially significant disruptions in labor markets and associated socioeconomic difficulties. Food production, like many other industrial sectors, has also undergone a century of mechanization, having moved toward increasingly large-scale monoculture production—especially in developed economies—with higher yields but detrimental environmental impacts on a global scale. Certain characteristics of the food sector and its products cast doubts on whether future automation will influence it in the same ways as in other sectors. We conceptualize a model of future food production within the socioeconomic conditions created by widespread automation. We ideate that despite immediate shocks to the economy, in the long run higher productivity can free up human activity to be channeled toward more interactive, skill-intensive food production systems, where communal efforts can reduce industrial reliance, diversify farming, and reconnect people to the biosphere—a realization of human well-being that resembles the classical philosophical ideal of Eudaimonia. We explore food production concepts, such as communal gardens and polyculture, and the economic conditions and institutions needed to underwrite them [e.g., a universal basic income (UBI)]. However, arguments can be raised as to why social-ecological systems would benefit from more labor-intensive food production. In this paper we: (1) discuss the current state of the food system and the need to reform it in light of its environmental and social impacts; (2) present automation as a lever that could move society toward more sustainable food production; (3) highlight the beneficial attributes of a Eudaimonian model; and (4) discuss the potential challenges to its implementation. Our purpose is to highlight a possible outcome that future research will need to refine and expand based on evidence and successful case studies. The ultimate aim is to promote a food system that can provide food security while staying within the safe operating space of planetary boundaries, produce more nutritious diets, enhance social capital, and reconnect communities with the biosphere.

## Introduction: the social and environmental problems of the modern food system

The twentieth century saw one of the most fundamental overhauls of the human food system since the widespread adoption of agriculture: this mid-late twentieth-century change is commonly known as “the Green Revolution.” An intensification of an already century-long application of modern scientific and engineering methods to farming, this revolution boosted yields by making use of improved genotypes, mechanization, pesticides, irrigation, inorganic fertilizers, and fossil fuels (1). As a consequence, food prices have reached historical lows, and larger caloric and protein intakes have increased life expectancies across most of the world (1). However, the negative consequences of large-scale intensive monoculture have also been conspicuous, with the modern food system exceeding four “planetary boundaries”; namely: biodiversity, climate change, land system change, and biogeochemical flows (2). Current food production is thereby undermining humanity's “safe operating space” on Earth. In addition, obese individuals today outnumber those undernourished (even though the latter still remains significant) (3), resulting in high morbidity and imposing large burdens on public health-care systems (4). The modern food system has also undermined modern medicine, through the overuse of pesticides and antimicrobials, which has led to a marked increase of antibiotic-resistant pests and pathogens (5). Therefore, producing more food on a smaller area, while using fewer resources, pesticides and antimicrobials, is one of the most pressing challenges for future food production.

More broadly, concerns and criticism over the prerequisites for economic growth with finite resources have also been mounting (6–8). Cheap fossil fuels that historically boosted economic growth will eventually dwindle, the monetization of past unpaid work may become saturated, and the regulations restricting polluting industries are likely to increase, with the likely consequence of slower economic growth rates in industrialized countries (9). In addition, mounting criticism over the dominant neoliberal economic growth paradigm has focused on its fixation on “more” instead of “better,” the privatization of public goods, and the promotion of globalization without regard for local social and environmental consequences (10, 11)—all of which connect to the prevailing critique of the industrial food system. The detrimental effects related to our current food system include: rapid population increase, economic growth as the primary policy goal, consolidation of the food system by large corporate and national players, commodification of food, increased marketing and advertisement leading to overconsumption, and the ineffectiveness of regulations at institutional, national, and global scales, among other things (12).

Mitigating the detrimental effects of the current food system while meeting future food security calls for a fundamental restructuring of global food systems (2, 13–15). Indeed, “food systems have to undergo radical transformations” (16) in order to align socioeconomic goals with environmental requirements in the future. The scientific community has suggested solutions, such as ecological intensification, agroecological farming, diversified farming, agroforestry, integrated farming, conservation agriculture, mixed crop and livestock, and other novel approaches (17, 18). Several “bottom-up” civic movements have also emerged in developed economies as a response to the overly instrumental, consumption- and production-maximizing ethos of the modern food system. Among these are organic foods, the “hundred-mile diet,” and farm-to-table agriculture, which in turn are fueling alternative food production systems, such as aquaponics, hydroponics, permaculture, backyard allotments, and urban gardening. These increasingly widespread practices promote food quality and diversity. Meanwhile quantity and convenience remain hallmarks of the current food system, with consequences for both health and environment (2). Unfortunately, food contributions from these novel, alternative systems are still marginal and confined to the affluent parts of the world. Most food procurement around the world remains driven by availability, affordability, and convenience (19). A change of these drivers in the near term is a remote prospect due to social, economic, and political inertia, caused by factors like the consolidation of food systems and the low willingness-to-pay for more sustainable produce (generally only around 10% more) (20). It has consequently become increasingly clear that shifting the food system to a more sustainable path requires qualitative socioeconomic changes, even while the political and public motivation to do so remains in short supply.

Therefore, we envisage that countries and communities—and particularly those already affluent economies that possess a certain set of advantages and difficulties—should use already unfolding socioeconomic transitions to catalyze a shift in food production and consumption. One such transition that could provide the technological and economic foundations for a fundamental restructuring of global food systems is the rapid trend of automation. As many jobs are likely to become redundant, we see the possibility for a transition of individuals toward a modern food production system that engages in meaningful communal endeavors. This food system would not only be less impactful on the environment, but would also improve social capital. Automation could guarantee a sufficient supply of food through direct engagement with food production (meeting the “quantitative” requirement) while freeing up human activity to pursue healthy and meaningful activities that increase well-being (raising the “qualitative” standard). We have termed this model of future development a *Eudaimonian* food system, in reference to the ethical ideal, enunciated by philosophers, such as Aristotle, of “human flourishing” based on connections with other people and with the natural world. Currently, the benefits of automation remain confined to the more affluent parts of the world, and may continue to be so for the immediate future. Corroborative institutional changes will consequently be needed to scale them up to the global level—a process that may take decades if not generations. However, we ideate that in the long run, such a transition will be necessary to achieve the Sustainable Development Goals (SDGs). In this paper we conceptualize the pathways of that transition and the potential benefits awaiting us with its completion.

## The catalyst for transition: automation

Automation is driving a major societal transition that is becoming increasingly conspicuous in many economic sectors, including food production. It is powered by technological investments in, and deployment of, machine capital to augment or replace manual labor. Automation is also an inevitable process with uncertain consequences. One concern that immediately arises in its wake, however, is rising unemployment and the undermining of social commitments to certain human rights (21). The consequent questions are therefore: “how redundant employees with no income will provide for themselves” and “how their absence from the labor market will impact an economy that is reliant on consumer demand” (22).

### Automation: historic trends and modern-day consequences

Automation is overtaking an increasing number of manual labor tasks and thereby making many occupations redundant or outmoded. A recent report concluded that adapting current technologies has the potential to automate about 50% of all paid labor in the global economy (worth an estimated US$15 trillion annually)—which is disproportionately concentrated in countries, such as China, Japan, India, and the United States—and in certain activities, such as data collection and physical labor (23). Based on work tasks across 800 occupations and using current technologies, the same report estimated that automation could fully replace 5% of those occupations, and that another 60% of those occupations could have at least 30% of their work tasks automated. Another report from the White House stresses that 47% of U.S. jobs are at risk of being replaced by AI technologies and computerization in the coming decade (24). The introduction of automated vehicles, impelled by safety and infrastructure benefits, could alone eradicate ≈3% of all U.S. job positions in a relatively short period of time (25). Automation of the retail industry, which is the largest sector (distributive trades) in the European Union, is in the meantime already underway (26).

Historically, rising unemployment often goes hand-in-hand with rising productivity and profits, in the agricultural sector as well as in the overall economy. Karl Marx famously deduced that a “reserve army” of displaced workers would be the moving force for political revolution, creating a new social order in which employment and income equality would be guaranteed for all. In the twentieth century, many non-Western countries engaged in the process of industrialization had to contend with such concerns. For instance, Japan's rapid economic growth in the first several postwar decades was in large part premised on transitioning large numbers of workers from the labor-intensive agrarian economy to the more capital-intensive industrial economy. In order to obviate mass discontent and potential political and social instability, it also invested heavily in worker retraining and the development of a large social safety net. As a result, the transition was overall a smooth and successful one, and Japan became a fully industrialized economy with a primarily urban high-income population (27, 28). Today, only 3.7% of Japan's workforce remains employed in the agricultural sector (29).

**Figure 1:**
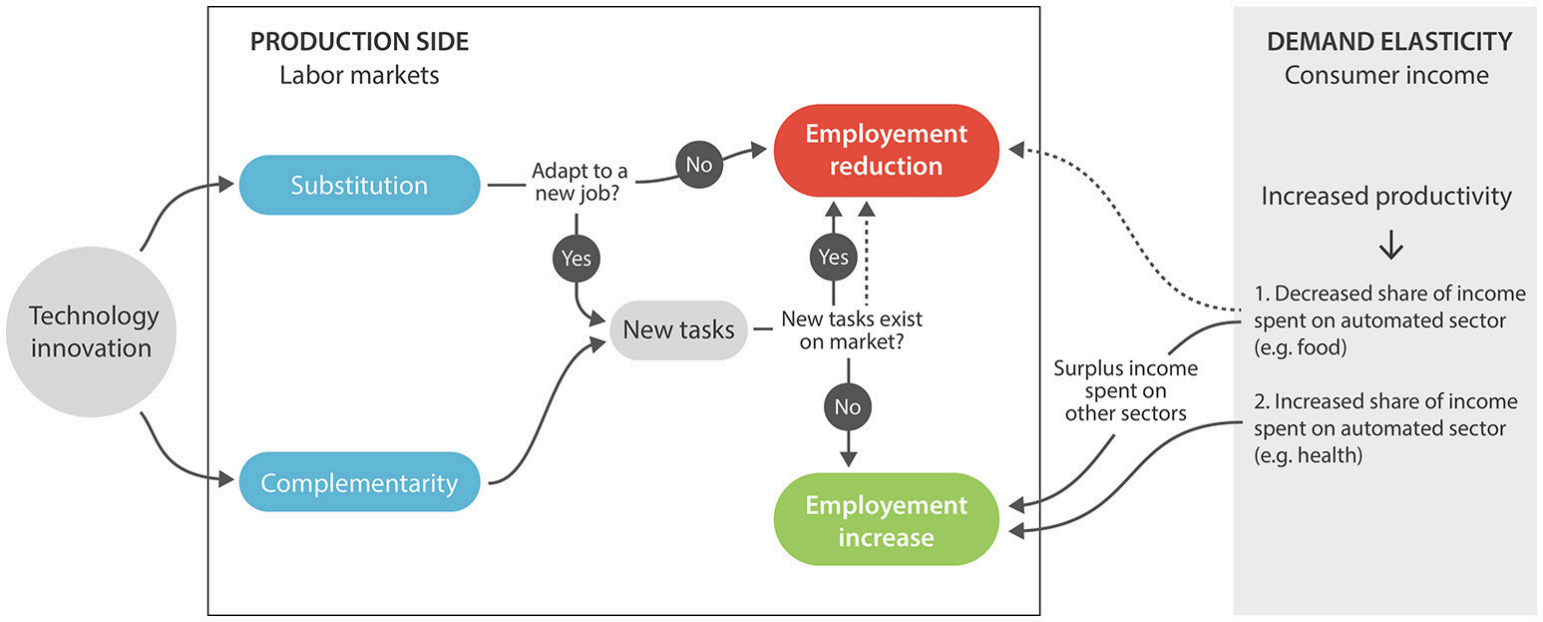
Technological innovations' impact on employment from production- and demand-side perspectives (left- and right-hand side, respectively). The figure is based on a qualitative analysis in Autor (30). Automation's impact is complex and can lead to both employment reduction (top middle) and increased employment (bottom middle) via creating of new economic niches or strengthening existing ones. Automation can substitute jobs (upper left) and result in unemployment unless the replaced workers learn a new job skill. In many cases automation and skilled labor can complement (lower left) increasing production and efficiency. New tasks can increase the amount of employment (arrow going down) or reduce it (arrow going up) depending on whether or not they are available elsewhere in the economy. New employment opportunities due to automation that are not available elsewhere (new niche markets) might not offset the loss of jobs replaced, resulting in overall reduction in employment (dashed arrow on the left going up). Automation can potentially increase efficiency and reduce economic expenditures, therefore reducing consumer demand and subsequently employment (dashed arrow on right-hand side), but overall household demand in goods and services has only increased in the long run, enlarging the economy and creating jobs. In some cases automation has increased household expenditure (demand) on certain sectors (e.g., health), which increase employment in that sector. Despite the complexity of the pathways detailed above that can potentially lead to increased employment or unemployment, historically aggregated employment has generally risen despite large changes in type and quality (30).

Changes in labor markets due to automation depend on multiple factors from both the supply and demand sides (Figure 1). Automation can lead to unemployment if the substituted workers' primary occupation is directly replaced, but jobs that are not directly substituted by automation are often complemented by it, increasing their demand and value (30). Therefore, although automation directly replaces labor, it doesn't necessarily result in reduced aggregated employment, at least in the long run. Because some sectors are more prone to automated replacements than others, there has been a polarization of labor markets in many advanced economies, with reductions of middle-skilled jobs and increases in high- and low-skilled employment (30). However, this polarization might not persist in the future because middle-skilled jobs increasingly require a combination of skills to perform routine and non-routine tasks, with the latter difficult to replace. Future automation might encroach on highly abstract, creative, high-skilled jobs as machine-learning unveils our tacit knowledge (30), but it should nonetheless be harnessed to replace routine jobs and leave the more fulfilling jobs to human laborers. Manyika et al. (23) state that the magnitude in shifts in the labor market due to the current wave of automation will be similar to the historical long-term shift away from agriculture and decreases in the manufacturing share of employment. The latter trends did not unleash large unemployment because they introduced new economic niches and opportunities that were unpredictable at the time.

Many proponents of automation, particularly in terms of the most recent wave—characterized by the deployment of robots, drones, and artificial intelligence—have argued that workforce disruptions and polarizations are perhaps regrettable, but also ultimately inevitable and beneficial, being part and parcel of economic development (23). This is in line with the economist Joseph Schumpeter's influential arguments about capitalism's motive force of “creative destruction” (31), in which new technologies and other innovations—including those that result in large-scale unemployment (i.e., “labor-saving”)—in the long run contribute to social progress. However, the time lag between the immediate impacts of labor market disruptions and the broader, ultimate benefits of increased productivity can leave room for the kinds of political and social instability warned of by analysts, such as Marx, and which were taken to heart by governments, such as Japan's during its process of economic modernization. As a result, many policies have been proposed, and some adopted, to forestall or ameliorate these problems. In addition to large-scale public investment in worker retraining, as was done in Japan, a universal basic income (UBI) (as will be discussed subsequently) has become increasingly advocated as an indispensable social policy (32, 33).

### The impact of industrialization and automation on food production and nutrition

The industrialization of the food system over the last century in the form of economies of scale, increased efficiencies, technological innovation, policy and globalization—all related in some way to automation—has had overarching impacts on agriculture production and consumption patterns (1). These global changes contributed to food security through increased food production, leading to more, longer, and healthier lives—particularly in impoverished countries that have struggled with subsistence (1). However, the focus has been on volume, resulting in adequate amounts of calories produced, but economic, social, and political factors still restrict access for many people around the world (34, 35). The Green Revolution, propelled in large part by technology, automation, and fossil fuels, has therefore allowed our current global population to grow in excess of 7 billion people.

Globally, this increase in availability triggered changes in food consumption patterns through large shifts from traditional diets to diets with increased animal products, processed foods, and low fiber (36). As nations become wealthier there has been an increase in the consumption of both more nutrient-rich food items (e.g., fruits) and low-quality foods (e.g., processed foods) simultaneously (36). In many countries, access to high-quality foods is largely reserved for the rich, while the poor consume mass-produced, nutritionally-depleted foods (ultra-processed foods), mostly derived from monoculture agriculture. Unequal access to animal proteins and healthy foods, in concert with changing dietary patterns (dietary transition), is further pushing people into malnutrition (35). Malnutrition issues have changed from widespread shortages to a more complex global pattern that includes increasingly imbalanced and excessive consumption. While at an all time low, the total share of undernourished people worldwide was still 10.7% of the global population in 2015 (29). More worrying, however, is the increasing frequency of malnutrition (4). Malnutrition includes undernutrition (i.e., wasting, stunting, underweight), but also inadequate access to vitamins or minerals, being overweight, obesity, and resulting diet-related, non-communicable diseases (37). Put in proportion to the 462 million people who are currently underweight worldwide, 1.9 billion adults are overweight or obese (3). Consumption patterns vary by regions and income level, but today the double burden of undernutrition and obesity coexist simultaneously, creating novel global health challenges and requiring effective interventions (38).

In recent decades, there has also been increasing attention to the environmental and social consequences of food system industrialization and what can be done about them. It is now undeniable that the industrialized food system causes detrimental impacts to the biosphere (2). Global food production requires widespread and intensive land, water, and fertilizer use, especially for the consumption of animal-sourced foods (13, 15, 39–41); this imperils the structure and services provided by global terrestrial and oceanic ecosystems (14). Furthermore, in recent decades, driven by increased economic efficiency, food systems underwent vertical and horizontal consolidation by a limited number of actors throughout the supply chain (e.g., seed distributors, wholesalers, and retailers). This concentration has shifted power and influence into the hands of a few players, marginalizing small-scale producers and disconnecting consumers from local food supply chains on a global scale. Consequently, consumers are now increasingly more reliant on exogenous food suppliers that mediate and influence their eating habits (42). Development has also been toward modern, high-yielding crop varieties (1). While this has limited the need for new farmland, sparing wilderness, and allowing for the maintenance of ecosystem services, farmers have in the meantime became more dependent upon anthropogenic inputs of chemicals and energy (1, 43). Modern high-yield monoculture is also more likely to cause other environmental consequences, such as soil degradation and aquifer depletion, and has resulted in a loss of the diversity of locally adapted strains and choices among consumers (1).

As part of the industrialization of the modern food system, the agricultural sector has been subject to automation, with tractors and other machines replacing human labor in developed countries during the twentieth century. In the United States, farmers' labor force has plummeted from 40% to about 2% of all labor (44). These trends and conditions are also true for much of the rest of the developed world, where manual labor in the agricultural sector has reached minimal levels. In coming years, large-scale automation through new technologies, such as automated harvesters and drones, is also likely to have significant long-term consequences for food production and distribution. This will undoubtedly exacerbate unemployment challenges through labor market disruptions. Although this will affect both developed and developing countries, the latter are likely to be especially hard-hit (23). In India, 44% of the labor force is still in the agricultural sector, while in Indonesia it is 31% (29). By comparison, the corresponding figures for the work forces of the European Union and the United States are around 1.5% (26, 45). Labor force disruptions could be precipitous, not gradual, especially if automation in food systems quickly reaches economies of scale and the prices for machinery and technology drop suddenly. This was, for instance, the case with solar panels, as a surge of large-scale investment (mostly in China) led to an unexpectedly sharp drop in prices over the past decade (46).

Already, some of these disruptions are beginning to be felt. Countries with the most “modern” workforces—i.e., those that have mostly shifted out of informal and agricultural activities into manufacturing and services jobs—and where food production and distribution are most industrialized are likely to experience the highest and most immediate economic benefits from automation (23). Here, automation can increase the productivity of workers—acting as complements rather than as substitutes—and offer a potential stimulus to flagging long-term economic growth rates. Already, drones have proved themselves as effective tools for crop monitoring (e.g., for pests and pathogens), crop spraying, and soil and field analyses (47). Coupled with advances in artificial intelligence, which is also experiencing a rapid increase of investment and application, productivity could be even higher (23, 47). These gains will help ensure the baseline of necessary food production needed to free up workers' time to engage in other activities (provided they also receive a UBI)—including the Eudaimonian social patterns that will be discussed in greater detail subsequently. In advanced economies with high labor wages and declining work forces such types of automation are already being widely deployed. However, for more agrarian economies over the short and medium terms, the upshot is much less clear.

## The requirements and priorities of eudaimonian food systems

In this paper, we envision that despite disruptions to labor markets, an increasingly automated global economy presents a unique opportunity for a transition to a more sustainable food system. We propose that the new economic order can be harnessed to reconfigure the food system to deliver increased food security, resilience, and reconnect individuals within a society to each other and to connect societies back to the biosphere (48). While acknowledging that different parts of the world are at different stages of economic development, with corresponding discrepancies in food systems, and that current trends in automation could be disruptive, we nonetheless project a future scenario in which long-term secular trends in technology and economics create the global capacity for what we describe below as a Eudaimonian food system. This future may not be achievable for all countries and populations at the same time, and as a planetary outcome could indeed be far into the future. Nonetheless, we believe that the model we propose below is an instructive starting point for an informed discourse on what constitutes a truly sustainable and ethical food system, and on the strategies and policies that could eventually get us there.

### Universal basic income and new employment patterns

In response to these concerns, the idea of a UBI has gained traction. The general concept of UBI is that each citizen would receive an unconditional regular transfer payment without means testing or work requirements (49). Since being first conceptualized in 1516 by Thomas More in his social and political treatise *Utopia*, UBI has become more of a plausible future reality than an idyllic dream—at least in developed economies with a welfare state tradition. Finland is, for instance, running randomized trials to evaluate the potential of this concept, driven in part by counterproductive rules related to having a part-time job while receiving housing allowance and/or social assistance (50). Additional arguments for its implementation have been to simplify complex social welfare systems, reduce inequality, intensify innovation, or, more radically, eradicate low-skilled and unfulfilling jobs (51). Other solutions to tackle contractions of the economy and social instability brought about by automation include work sharing, increased social security, and alternative economic niches (10). To accommodate more employees, work sharing includes the employment of more people for fewer working hours, during which economic productivity is replaced by higher social and personal benefits (10). In addition, a strengthened social security system will offer a safety net to the unemployed in the form of a basic income and a public job-providing system for those in need.

**Figure 2:**
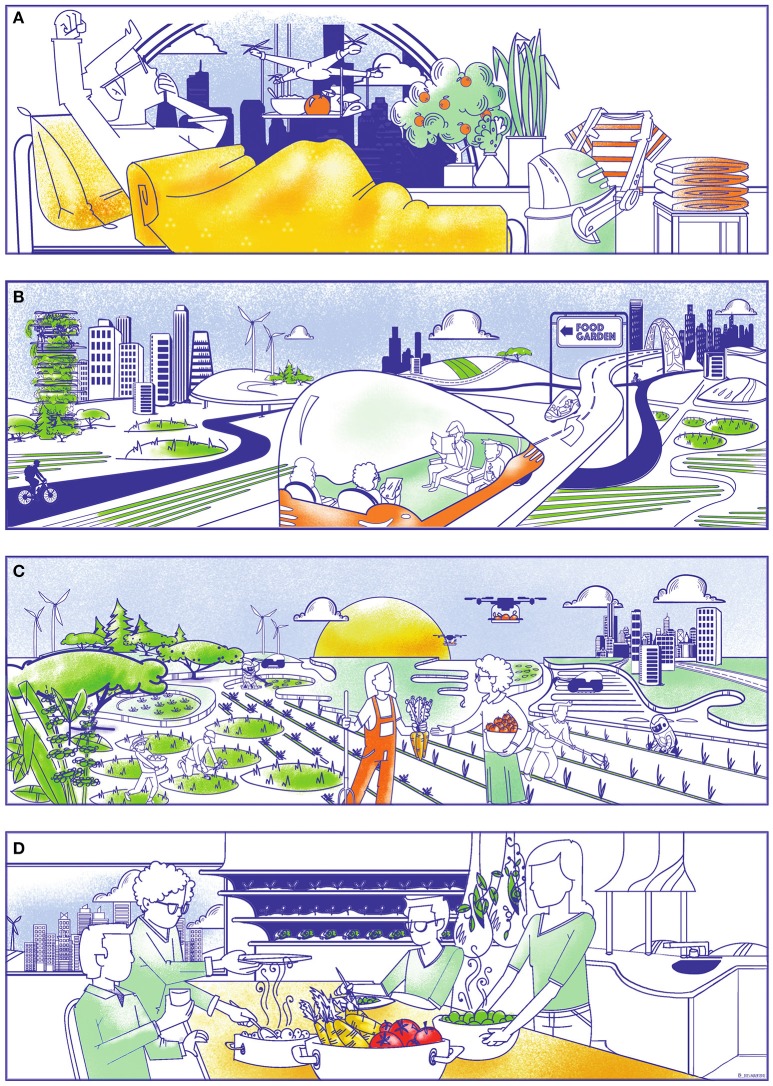
A day in a Eudaimonian food system (from top to bottom). **(A)**: 9 a.m.: Given greatly reduced or even non-existing working hours since the introduction of UBI, the need for alarm clocks has been greatly reduced. Many household chores have also been automated, leaving citizens with more spare time. **(B)**: 11 a.m.: Traveling to gardening spaces together with friends or community members in automated vehicles. The transition to automated vehicles has also opened up parking spaces for gardening, restaurant terraces, bicycle lanes, and other activities. **(C)**: 4 p.m.: After half a day's work in the community garden, the daily harvest is shared and discussed. Labor-intensive and redundant tasks have been automated, to leave the most rewarding tasks to the gardener. Weeding and pest control have also become mechanized, further reducing our reliance on chemicals. **(D)**: 7 p.m.: The farm-to-plate concept is no longer a luxury of a few restaurants or rural citizens, but commonplace during dinner parties where crop varieties, farming practices, and preparation techniques are frequent topics for discussion. Seeds are commonly exchanged and plant sprouts prepared in the house. Time investments and simplified distribution chains have also resulted in a great reduction of food waste, where produce is selected for its flavor, not its productivity, nor its esthetics. Design by Iris Maertens (www.irisistible.design).

The disruptions and shifts in labor markets that will result from automation are the driving force behind the transition to the Eudaimonian system we envision. There will be a need for new economic niches that require high-skilled jobs, and with UBI and work-sharing mechanisms, a new market niche, which we term “Eudaimonian farmers,” can be created (Figure 2). UBI bestows a safety net protecting these new agriculturalists from economic insecurity, which is commonplace among current farmers (whether in developed or developing countries, farmers often operate on razor-thin margins of profitability, and are especially vulnerable to price fluctuations in the global markets); work-sharing is a mechanism that allows the dividing of physical and mental burdens among many participants, thereby reducing the stress and difficulties befalling individual full-time farmers. Working reasonable hours in diverse agroecological systems (e.g., open field polycultures, diverse urban settings), these new farmers can produce food while reaping multiple benefits both at the individual and community level. To be operational, successful, and able to produce ample food, this new economic niche will require policies and institutional support as we detail below.

### Agroecological food systems

We envision the Eudaimonian food system to be based upon diversified agroecological food production systems (52–54). This is contrary to the current industrial system, which has emphasized increased trade, commoditization of food, and large-scale production through uniformity (monocultures). Agroecological systems are diverse food production systems that mimic natural ecosystems and deliver—apart from edible food—multiple ecosystem services taking into account economic, societal, and environmental dimensions (52, 54, 55). These ecosystem services include flood regulation and clean water, carbon storage and soil preservation, biodiversity conservation, air purification, recreational amenities, and more.

These diverse agroecological systems range in structure and crop assemblage based on climate, biomes and people, but encompass universal attributes, such as high complexity, multiple outputs, polyculture, short value chains, nutrient-rich landscapes, and increased food sovereignty (52). Due to their diversity and non-homogeneity, agroecological systems are usually more labor intensive, but this is not always the case and can be improved through efficient labor organization and technological innovation (56). Successful management of these systems requires time for adaptation and learning. The nature of labor itself also changes, requiring new sets of skills and knowledge (56). The erection and maintenance of these complex agroecological systems will require highly skilled labor, and will not be easily replaced by automation. These “new” agrosystems thus serve as a new market with the opportunity to absorb a surplus workforce while creating socially and personally rewarding activities. Resembling diverse natural systems to some extent, these landscapes hold greater aesthetics, and thus foster broader reconnections to the biosphere (57). They would naturally also reward participants in the form of a wider variety of fresh and safe produce.

### Food sovereignty

The concept of food democracy was first introduced as a force that shapes the food system through the participation of many in the interest of the public good. This stands in contrast to “food control,” the paradigm of a food system that is “top-to-bottom” controlled and shaped by a small number of actors (58). Food democracy asserts the need to democratize the way food is produced and consumed and dissolve the concentration of market power by increasing the role of citizens in managing and controlling the food system (often termed “food citizenship”). First coined by the international farmers' movement Via Campesina (59), food sovereignty emphasizes the same principles of food democracy only with greater weight on human rights and small-holder farmers (production-side) (60), thus advancing a food system based on equity and justice and fair access to resources. The current emergence of proactive “civic food networks” in the form of co-ops, community supported agriculture, and community gardens entail socioeconomic ramifications that are increasingly affecting the entire food system (60), serving as case studies and demonstration sites to the transformation we describe here. With its emphasis on short and local supply chains, agroecological practices, citizen participation and civil governance, the Eudaimonian food system contains the essential conditions of food sovereignty.

### Social unity and trust

As detailed above, we believe the model we outline will foster greater food citizenship, increase community cohesion, engage individuals with agriculture and nature, and enhance consumers' sense of place (61). Current communal gardening practices—with similar attributes to our Eudaimonian food system—demonstrate the social benefits that can be reaped in terms of promoting a sense of social belonging and individual well-being (62, 63). Local food systems prioritize community, build local leadership capacity, and serve as defenses against certain economic, cultural, and environmental vulnerabilities, contributing to community cohesion (64).

Social capital is thus a fundamental component in the future food system we outline. The success of these societal changes is largely dependent upon the trust in the government by citizens (65), and is thus most likely to be successful in high-income countries with low inequality—which are, admittedly, rare in the current world, but which nonetheless constitute generally accepted development goals. Trust in the government is also correlated with a higher willingness to pay taxes, a prerequisite for a society with UBI. In addition, short and local food supply chains establish greater trust and value (66). A Eudaimonian food system where a greater number of citizens participate in food production builds better community capacity and strengthens the local community through increased food security and more affluent local economies.

### Safeguarding cultural and natural heritage

Food has a central role in our cultural heritage. Sustainable Development Goal 11.4 urges the duty to “strengthen efforts to protect and safeguard the world's cultural and natural heritage.” Food ties local space and culture in many ways (66), establishing “culture economies” (67) and “foodsheds” (68). Localized food systems as proposed here can serve as places where community, landscape, and culture interact (66).

Unlike cars or smart phones, where automation may allow for a few great products at more affordable prices, diversity is central to an attractive food system from gastronomical, nutritional, and biodiversity perspectives. The French-derived term *patrimonialization* defines the confluence of authenticity and prestige of food in the context of regional cuisine, the protection of rural landscapes, and the value of food products linked to specific land spaces (69). Contrary to conventional food production systems, such a food production system could act not only to reduce our reliance on external inputs, but also reconnect the public with natural and rural landscapes and their biodiversity, reinforce resilience, and protect cultural values (2, 70).

Wines, beers, cheeses, and bread are examples where diversity is actively rewarded by premium prices and high cultural identity and prestige. Some cultures seem to build their culinary fame on a diversity of local and regional delicacies—from North America to Europe and Asia. In contrast, the adoption of modern agricultural practices easily results in socio-ecological traps and a loss of diversity (70, 71). It could also be linked to a loss of cultural belonging, as can be exemplified with potato varieties in the Andes or tomatoes in Southern Italy (71). Bottlenecks for plant diversity commonly occur at the initial domestication of the cultivar and during the selection of modern elite strains (72). While limited data exists on the historical genetic diversity of different crops, there has been strong consolidation within the seed industry and concerns of loss of diversity had initiated projects, such as the Global Seed Vault in Norway. Apart from the loss of genetic diversity, there has also been a loss in culinary experiences. This has left farming enthusiasts and gastronomes to form informal networks, such as the Seed Savers Exchange. Reforming local sustainable food systems that partially replace globalized, long chain food systems stands to affirm a greater cultural identity, reconnecting the local population to their cultural tradition and bestowing a greater sense of pride in their activities. It could also help preserve a greater diversity of strains, which will add resilience to future challenges, such as climate change.

### Feeding into the sustainable development goals

The Eudaimonian system we present contributes to many of the 17 Sustainable Development Goals (SDGs) promulgated by the United Nations, and provides enhanced food security. The system is based on agroecological principles, reducing inputs, preserving soil fertility, clean water and air, and maintaining biodiversity (SDG Goals: 7, 13, 14, 15). The complexity and diversity of these agrosystems in the form of polyculture, vertical farming, and other practices produce diverse, healthy, and high-value foods (SDG Goal: 3), which stand in marked contrast to the products of monocultures. The sustainability of the food system is ensured throughout the entire supply chain (production to consumption) through consumer's direct participation in food production and short supply chains, which reduces food loss (SDG Goal: 12). Food sovereignty (section Food Sovereignty) is an integral part of the food system we propose, ensuring the right of all citizens to nutritious and healthy food, alleviating food poverty and hunger, and contributing to reduced inequalities. Contributing to SDG goals 1, 2, 3, 5, and 10, this component is fundamental in providing food security by increasing access and utilization. The direct participation in producing food contributes to human well-being via physical activity and direct linkage to the biosphere (SDG Goal: 3), fostering a lifestyle that includes better health and sense of community (section Social Unity and Trust). Given that food production is no longer exogenous, traditional and cultural preferences in food choices can be readily respected (section Safeguarding Cultural and Natural Heritage). To implement and operate the Eudaimonian food system will require adequate food polices and strong institutions (SDG Goal: 16 and sections Safeguarding Cultural and Natural Heritage, Relevant Policies), forging greater ties and partnerships in the long run (SDG Goal: 17 and section Support Across Sectors and Agents). Table 1 summarizes the main attributes of the new food system we introduce and compares it to the prevailing industrial food system.

**Table 1:** Characteristics of the industrial and the Eudaimonian food systems.

	**Present industrial food system**	**Eudaimonian food system**
Food citizenship	Limited and difficult to ensure	Available to all
Biodiversity	Industrialized agriculture still heavily relies on monocultures, dominated by high-yielding varieties	Rich in species, functional traits, and genetic diversity
Chemical use	A general overuse of inorganic fertilizers and pesticides in many regions of the world	Pesticide use limited by self-preservation and biological control. Nutrients through household composting
Energy use	Mainly fossil fuels	Human labor and electricity for automated tasks
Community belonging	Decoupled from food production	Strengthened by the food production system
Safety nets and support	Limited	Government and civil society supported (section Support Across Sectors and Agents)
Food equality	A correlation between poverty and malnutrition	Citizens living on UBI and equally participating in food production
Addressing the Sustainable Development Goals	Few	Many of the goals
Resilience	Vulnerable to climatic events, disease, and physical or monetary disturbances to the flows of food, fuel, or inorganic fertilizers	Enhanced through a reduced reliance on anthropogenic inputs and increased crop and genetic diversity
Ecosystem services	Few	Many
Connection with the biosphere	Low	High

### Urban agriculture in an urbanized world

Cities are an ideal location where the Eudaimonian food system we propose can be implemented. Currently, half of the global population resides in urbanized landscapes that cover <1% of total land area. The share of people living in cities will continue to grow over the coming decades, with little sign of abatement. Efforts to increase resilience, adaptive capacity, and food security in these places will provide large benefits as they impact a disproportionately large fraction of global populations within relatively small land areas (73). Nowadays community gardens, peri-urban agriculture, vertical farming, and rooftop cultivation are just a few examples showcasing small-scale food production at heterogeneous locations within urban settings. With urban population poised to increase substantially in the upcoming future (74), urban agriculture lends itself to increasing food security while partially alleviating the geographic extent of hinterlands for food production. The role that urban food production will play in future food provision is an ongoing research effort (74), but increasing food production within cities is an important strategy to increase ecosystem services (74), increase food citizenship through participation, increase food diversity (e.g., eliminating “food deserts”), reduce food mileage and loss, and increase the stability of supply (another dimension of food security). In addition, urban agriculture has been recognized for its social, ecological, and economic virtues. Gardening bestows therapeutic values via physical activity and spiritual and emotional renewal, contributing to health and well-being (75). Urban agriculture strengthens communities and increases social capital (74). The proximity of food production sites, residents (consumers and work labor), and governing institutions reduces logistic and supervision burdens and enables upscaling and streamlining of food systems in cities.

## The challenges facing the achievement of eudaimonian food systems

The Eudaimonian food system we ideate faces many challenges in implementation and upscaling. Mier y Terán Giménez Cacho et al. (76) identified key drivers for upscaling agroecological systems using case studies from around the world. These include: a crisis that challenges the current system; social organization (belonging to a network/community); comprehensive and abiding discourses; education processes that disseminate knowledge across farmers; allies from government, academia, and NGOs; a media that supports, lobbies and promotes; favorable markets that create an alternative food network; and favorable policies. These driving forces are consistent with what we describe in the previous sections: automation (*the crisis*) creates the backdrop upon which reforms to the current industrial food system can be installed. Construction of a mass of people (*community/movement*) engaging in agroecological practices to produce food for consumption (*favorable markets*) supported by protective policies. We list below some of the main challenges to implementing a Eudaimonian food system.

### Engagement and motivation

Education, especially from an early age, will play an important role in facilitating the normalization of such drastic transformations of food production. Education at the family, community, and school levels are imperative to building trust (section Social Unity and Trust), responsibility, healthy lifestyles (e.g., regular physical activity), and healthy-eating norms—all prerequisites to adopting a Eudaimonian food system. In this context, effective policies (section Relevant Policies) and education in hospitals, workplaces, and schools are instructive in promoting a transition in the way people perceive food and health (77). The school environment is an ideal setting to bring about changes in behavior, consciousness, and skills related to food through focusing not only on healthy consumption but also preparation, preservation, and storage of food (78), as well as self-production at school and educational gardens (79).

How do you motivate people in the developed economies to return to producing food? Insights into current farmer motivations can be constructive to some extent in devising policies and mechanisms that encourage people to work on a part- or spare-time basis, for non-subsistence or profit-driven reasons. The surge in organic agriculture production in the United States in recent decades provides valuable lessons to promote engagement in agriculture. Profit is an important objective but other non-economic motivations, such as environmental stewardship and lifestyle changes are also important, especially for the young (80). Peterson et al. (80) found that yield losses and the definition of organic markets were the major concerns expressed by farmers. In rural communities, reluctance to take on farming is a result of several factors, including downgrading the occupation of farming in formal education, lack of access to land for various reasons (especially in developing economies), and the social or cultural devaluation of manual work (81). In a study of dairy farmers, the motivation of people to engage in dairy farming is driven by factors that are specific to any workplace, such as pride, occupational meaningfulness, pleasure from the activity, job security, flexible tasks, and positive leadership, in addition to those that are more specific to farming, such as a countryside setting (82).

### Relevant policies

Policies that facilitate the transformation and oversee its operation are crucially important. Although automation injects large uncertainties into labor markets, redeployment will be a major societal challenge, requiring planning and interventions by governments (23). Apart from the shift in labor markets and the unemployment that needs to be addressed via redeployment programs and safety nets (including UBI), the need to restructure parts of the agriculture sector, allocate lands, set up regulatory mechanisms, and support relevant legislation will require overarching policies. Apart from redirecting the unemployed labor force, supervision and logistic coordination of the new scheme could be provided by government programs, civil society, or private sector organizations.

In this respect, Cuba may offer an instructive example. Its government has been instrumental in decentralizing food production on large state farms and promoting smaller urban plots run by individuals and institutions following the dissolution of the Soviet Union and the collapse of a once global network of socialist states (76, 83). Despite this deregulation, the government provided strong support through improving access to land, providing extension services, research and technological development, and marketing programs (83).

### Support across sectors and agents

The involvement of actors from other sectors including civil society entities, such as NGOs, universities, and the media is vital (76). Civil governance of food networks has been part of a growing trend of alternative food systems, challenging conventional mainstream food pathways, which are mostly controlled by the state and corporate interests in the market (60). Incentives to promote the private sector to take part in our proposed transition are also necessary. As an example, in Sweden, privately run job-security councils supported by large corporations have been instrumental in providing a vocational safety net and helping the unemployed find new jobs with exceptional success rates (84). It will be a challenge to chart future food policies that encourage a balanced mix of all these sectors while putting public interest above all.

## Discussion and conclusions

Our hypothetical future Eudaimonian food system and the possible changes it relies upon are based on a priori assumptions, highlighting a possible, but in many ways also necessary and desirable, outcome. We do not depict it as the only possible future reality—we present it as a scenario that is plausible in the long term, and hopefully also desirable (in that it addresses the outstanding social and environmental problems of the current food system). Thus, understanding the policy and measures needed to realize—and the potential benefits that result from—a Eudaimonian food system is a first step in exploring a viable alternative to the current food system, premised in large part on the prospects of automation. Future research and *in situ* case studies will help to refine the ideas suggested here and identify successful measures to embrace as well as pitfalls to avoid. We have discussed examples of both in the preceding sections.

Our work complements numerous calls to transform the current food system from an industrialized, consolidated system into one based on agroecological principles and food citizenship. Our main contribution is in identifying automation as a lever to achieve this shift. In this paper we propose harnessing the changes in labor markets brought about by automation to reshape the food system into what we refer to as a Eudaimonian system. By employing solutions, such as UBI and labor market-based mechanisms, such as work sharing, we can engage people to become part- or spare-time farmers, working in communal or private gardens without the economic insecurity or lack of support that are ubiquitous to farmers worldwide. It will also provide benefits through physical activity, community engagement, and reconnecting to the biosphere.

The Eudaimonian food system we envision relies upon people engaging in food production, a trend that runs counter to historical results of mechanization. Industrial agriculture, lack of policy and the dominance of corporations over the food system in recent decades have been the product of a neoliberal global economy in which business efficiency, privatization, and trade liberalization have been the driving forces, reducing the number of people engaged in food production and causing other disruptions to social capital. Increasing the number of participants in food production also goes against corporate interests, as it reduces economic efficiency in its current narrow definition (i.e., direct revenues vs. expenses). However, using a broader definition of economic development, one in which public expenses on health and environment are also included and a long-run horizon is taken into account, our proposed food system could not only be seen as practical, but also as necessary. This is true because in fiscal terms, the reduction of environmental impacts due to the current industrial food system (e.g., by internalizing externalities) and improvements in public health due to adequate and healthy foods will reduce large fractions of public expenses (future work will need to substantiate whether these savings are comparable to reductions in the direct efficiency of food production through increased labor). The added values in the form of social cohesion, food citizenship, cultural identity, and other attributes we highlight in section The Requirements and Priorities of Eudaimonian Food Systems are harder to quantify in monetary terms, but would surely advantage a Eudaimonian food system over our current food system.

An argument often raised in favor of the current, highly productive industrialized system and against reform relates to producing enough food to feed the continuingly growing global population, whose appetite for resource-intensive foods has not declined. While the food system we propose here will not feed the whole global population immediately, our study augments a large body of work that highlights the importance of agroecological food systems, why they are crucially needed, and how they can provide enough food to stave off hunger and malnutrition in the future. In addition, it is worth noting that the concept of food security goes beyond food availability (production) to include other important dimensions, such as accessibility, utilization, and stability. Current malnutrition and hunger in many countries is the result of many factors, and not solely, or often even primarily, because of insufficient food production. We believe that direct engagement in food production, higher transparency in the food system, strengthened cultural identity, as well as social capital will increase food security through more nutritious and diverse foods, reduced waste, communal cohesion, and ecological resilience.

Our belief is not that these Eudaimonian systems will produce staple crops, such as wheat or rice, nor that they would need to be fully organic, but that they will provide more palatable varieties of produce, increase diversity, and improve public health, while at the same time delivering on public welfare, community, and aspects of social justice. By addressing many of the drivers behind food selection, including health, environment, animal welfare, ethics, or cultural identity (35), a Eudaimonian food system would likely appeal to a broad subsection of consumers, especially since it would come without prohibitive costs. This would also improve health among those of lower socio-economic status, a group that would probably be reliant on the UBI and suffer from poor dietary options (35). It would also help solve food supplies for an increasingly urban world population and distribute nutrition more equitably (85). It could also help reverse the current pattern of one-way flows of nutrients in modern agricultural systems (85) by making composting available to more citizens. This would draw parallels with the UK government-initiated “Every Man a Gardener” campaign that supplied substantial amounts of fresh vegetables to urban populations during WWI, as international trade routes were severed (85). The more recent surge of alternative food networks in the form of urban agriculture, farm-to-table movements, and farmers' markets is a proof of concept that alternatives already exist. Streamlining these trends to include larger numbers of participants and produce larger quantities of food in a concerted and incentivized effort can be achieved with automation.

In 1930, during the Great Depression and amid rising geopolitical tensions, John Maynard Keynes wrote his famous essay, “Economic Possibilities for Our Grandchildren.” In it, he projected a utopian future in which, roughly a century hence, rising productivity and the accumulation of wealth will have overcome scarcity and made most manual work unnecessary (86). The challenge then, Keynes surmised, would center on the improvement of the quality of life, not the quantity of economic activity. Work, such as farming would be more of a leisure activity (to satisfy the promptings of the “old Adam in most of us”), not an essential task to maintain livelihood. Keynes's future scenario was ultimately wide of the mark: he was prescient about the gains in productivity and wealth the world would experience, but economic growth still remains the primary pursuit of countries around the world. A future in which people are largely free of economic burdens and cultivate what he called “the arts of life” and “activities of purpose” is still remote. We recognize the dangers of sweeping prognostication and acknowledge that some of the ideas presented here might seem unrealistic in today's society. Nonetheless, we believe growing automation can be harnessed to fundamentally reform our food production system, even if the results fall short of our proposed goals. Additionally, although Keynes's optimism was not fully vindicated, the scenario he delineated remains a touchstone in many discussions about the morality and sustainability of industrial economic systems (87). Future generations will surely look back at our industrial food sector in ways similar to how we now look back at the whaling industry, or other happily obsolescent socioeconomic conditions and environmental norms that prevailed a century ago.

## Author contributions

PH conceived the idea. AS and PH designed the study and were in charge of overall direction and planning. AS took the lead in writing the manuscript and supervising the project. All authors carried out the research and contributed to the interpretation of the results. All authors discussed the results and were involved in writing the manuscript.

### Conflict of interest statement

The authors declare that the research was conducted in the absence of any commercial or financial relationships that could be construed as a potential conflict of interest.
